# Type 2 diabetes mellitus as a possible risk factor for myasthenia gravis: a case–control study

**DOI:** 10.3389/fneur.2023.1125842

**Published:** 2023-04-17

**Authors:** Yu-Dong Liu, Fang Tang, Xiao-Li Li, Ya-Fei Liu, Peng Zhang, Chun-Lin Yang, Tong Du, Heng Li, Cong-Cong Wang, Ying Liu, Bing Yang, Rui-Sheng Duan

**Affiliations:** ^1^Department of Neurology, The First Affiliated Hospital of Shandong First Medical University and Shandong Provincial Qianfoshan Hospital, Jinan, China; ^2^Center for Big Data Research in Health and Medicine, The First Affiliated Hospital of Shandong First Medical University and Shandong Provincial Qianfoshan Hospital, Jinan, China; ^3^Shandong Institute of Neuroimmunology, Jinan, China

**Keywords:** myasthenia gravis, type 2 diabetes mellitus, case control study, diabetic myasthenia gravis, advanced glycation end products

## Abstract

**Background:**

A certain number of myasthenia gravis (MG) patients clinically had type 2 diabetes mellitus (T2DM) prior to MG onset, which suggests that the onset of MG may correlate with the history of T2DM. This study aimed to examine the correlation between MG and T2DM.

**Methods:**

In a single-center, retrospective, 1:5 matched case–control study, all 118 hospitalized patients with a diagnosis of MG from 8 August 2014 to 22 January 2019 were enrolled. In total, four datasets with different sources of the control group were retrieved from the electronic medical records (EMRs). Data were collected at the individual level. A conditional logistic regression analysis was used to test the risk of MG associated with T2DM.

**Findings:**

The risk of MG was significantly associated with T2DM, and there were notable differences by sex and age. Whether compared to the general population, general hospitalized patients without autoimmune diseases (AIDs), or patients with other AIDs except MG, women aged over 50 years with T2DM had an increased risk of MG. The mean onset age of diabetic MG patients was more than that of the non-diabetic MG patients.

**Interpretation:**

This study demonstrates that T2DM is strongly associated with the subsequent risk of MG and varies significantly by sex and age. It reveals that diabetic MG may be a unique subtype that is different from the conventional MG subgroup classification. More clinical and immunological features of diabetic MG patients need to be explored in further studies.

## 1. Introduction

Myasthenia gravis (MG) is a B-cell-driven, T-cell-dependent autoimmune disease (AID), which results from the transmission disorder in the post-synaptic neuromuscular junction caused by the production of antibodies for acetylcholine receptors (AChRs) (1, 2) or other proteins, such as muscle-specific kinase (MuSK) (3). Low-density lipoprotein receptor-4 (LRP-4) and agrin were also considered indicative of MG. The main clinical manifestations of MG include muscle weakness and abnormal fatigability which seriously affect the quality of patients' daily life. Generally, MG can be divided into different clinical subtypes. According to the different distributions of muscle weakness, MG can be divided into ocular MG (OMG) and generalized MG (GMG) (4). Grouped from the age of onset, MG patients also can be divided into early-onset (EOMG) and late-onset (LOMG) forms by the age of 50 years (5). It has been confirmed that the incidence of MG in the elderly of both sexes is increasing (5). Incredibly, very late-onset MG patients (onset age: 65 years or older) are more prone to severity (6). Thymoma-associated myasthenia gravis (TAMG) was considered a different subset from EOMG/LOMG or OMG/GMG clinical classification because of its specific characteristics in pathogenesis and clinical features (7). The traditional therapies of MG are acetylcholinesterase inhibitors (AChEIs), immunomodulatory therapies, thymectomy, and so on (8).

Type 2 diabetes mellitus (T2DM) is a common chronic disease, characterized by elevated blood glucose and attenuated insulin action. The incidence of T2DM has been rapidly increasing over recent decades, which has become a significant public health problem in the general population of China (9). Although the traditional view is that the correlation between MG and DM might be related to the adverse effect of corticosteroid and aggressive therapy (10), a 35-year retrospective study in 2007 raised doubts that it remained unclear whether prednisolone administration directly induced DM or if it merely accelerated disease onset (11). We found that quite a lot of MG patients had T2DM prior to the onset of MG. Traditional views cannot explain the phenomenon we found in the clinical work and may even obscure the true relationship between T2DM and MG.

In the present study, we propose a preliminary hypothesis: T2DM may be associated with MG. A matched case–control study was designed to systematically test the hypothesis. The chronological order of the onset of T2DM and MG was also considered. Furthermore, the correlation between T2DM and MG, potential clinical features, and possible molecular mechanisms were elaborated. This discovery will provide innovative ideas for MG diagnosis and treatment.

## 2. Materials and methods

### 2.1. Data sources

Data at the individual level were retrieved from electronic medical records (EMRs) in the First Affiliated Hospital of Shandong First Medical University between 8 August 2014 and 22 January 2019. The time of onset of MG and T2DM and the history of medicine compliance, such as glucocorticoid, were collected and recorded by tracking the patient's progress through EMRs and regular follow-up visits. Patients with ambiguous information were not included in the study.

### 2.2. Criteria of participants

A total of 141 inpatients (74 male patients and 67 female patients) with a diagnosis of MG were retrieved from EMR. The diagnosis of MG was made according to the following parameters (12): (1) the definite typical history and symptom of fluctuating weakness as well as fatigability of voluntary muscles; (2) the presence of serum anti-AChR antibody or other MG-associated antibodies (MUSK-Ab); (3) positive response to the neostigmine test; and (4) low-frequency repetitive nerve stimulation (RNS) reveals that the amplitude decreased by more than 10% (13, 14). Collected data included demographics, comorbidities (including other discharge diagnoses except MG), clinical type of MG, results of antibody testing, laboratory results, and electrophysiological data. The boundary age between EOMG and LOMG is 50 years. The diagnosis of TAMG is based on the patient's pathological diagnosis.

Participants were diagnosed with T2DM according to the 1999 World Health Organization criteria (15). The diagnostic information of MG or T2DM must be consistent with the records of discharge diagnosis or past medical history from EMR.

The inclusion criteria for MG patients included a clinical diagnosis of MG: (1) MG must be conclusively diagnosed based on the diagnostic criteria and (2) the onset age must be at least 18 years old. Meanwhile, the following were the exclusion criteria: (1) participants with other AIDs except MG and (2) diabetic MG patients who took glucocorticoids before the onset of DM, considering the possible effect of glucocorticoids on T2DM pathogenesis. The Global Autoimmune Institute (GAI) made a list of recognized autoimmune diseases available.

Overall, 23 patients were not included in the study due to the following reasons: one patient had an unclear diagnosis of DM; one patient's information was partially missing; 10 patients took glucocorticoids before the onset of DM; six patients had other AIDs; and five patients were <18 years old at onset. Finally, a total of 118 patients were included in the study.

### 2.3. Criteria of matched controls

**Figure 1:**
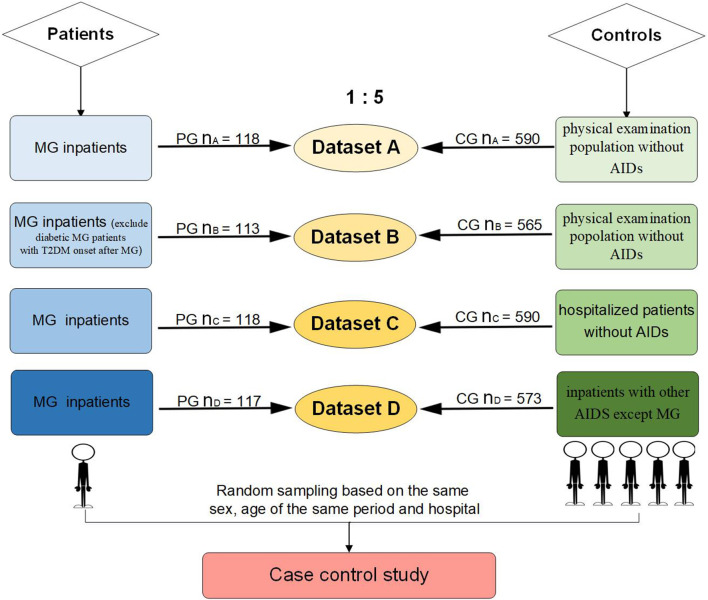
Flowchart of MG patients and controls inclusion (PG, patient group; CG, control group).

Up to five controls per MG patient, matched for sex and date of birth, were randomly drawn from people of the same period in the same hospital. A total of four datasets were analyzed (Figure 1): (1) Dataset A: 118 MG patients and 590 controls, which were randomly selected from a population without AIDs of the health examination system. (2) Dataset B: 113 MG patients and 565 controls, who were diabetic MG patients with a T2DM onset time after MG or an uncertain onset time of diabetes, and their controls were excluded from dataset A. (3) Dataset C: 118 MG patients and 590 controls, which were randomly selected from all hospitalized patients without AIDs. (4) Dataset D: 117 MG patients and 573 controls, which were randomly selected from inpatients diagnosed with AIDs except MG. In total, one MG patient was removed from the patient group of dataset D because she was too old (89 years old) to match the eligible controls.

### 2.4. Statistical analysis

Descriptive analyses were used for the distribution of variables of interest in the study population. Categorical variables were compared by the χ^2^ test. Continuous variables were compared by the *t-*test or non-parametric test. A conditional logistic regression analysis was used to test the risk of MG onset associated with T2DM. We stratify the particular MG case based on the onset age and sex in different MG clinical subtypes to eliminate the interference of confounding factors.

### 2.5. Ethics approval

All clinical information was obtained after the patients had given their written informed consent. The research received approval from the Research Ethics Committee of the First Affiliated Hospital of Shandong First Medical University (No: S030).

## 3. Results

**Figure 2:**
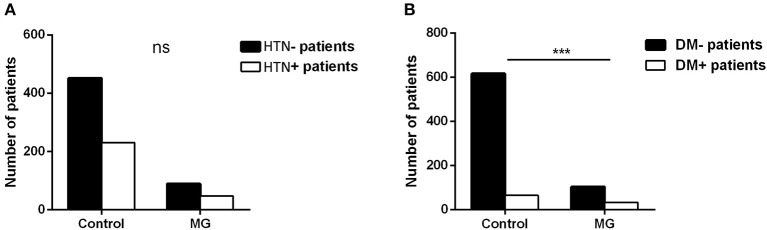
HTN and T2DM prevalence **(A, B)** between 676 control people and 136 MG patients (the controls of the same sex and age in the same period were randomly sampled from the physical examination system to match MG inpatients in the same hospital; *t*-test, ***P < 0.001).

Our study showed that the top eight comorbidities of MG with prevalence were hypertension (HTN) (34.04%), thymoma (27.66%), T2DM (23.40%), coronary heart disease (18.44%), pulmonary infection (16.31%), cerebral infarction (16.31%), pneumonia (7.09%), and cataract (7.09%). Most comorbidities, except for thymoma, were related to geriatric chronic disease or infection. The correlation between thymoma and MG is already acknowledged, and the other top two comorbidities (HTN and T2DM) were focused on. The prevalence of HTN and T2DM in the MG inpatients and the general population was separately collected and analyzed. In terms of the prevalence of HTN, there was no statistical difference between controls and MG patients (33.77% vs. 34.56%, *P* > 0.05), while the T2DM prevalence of MG patients was higher than that of controls (24.26% vs. 9.54%, *P* < 0.001) (Figure 2).

### 3.1. Prevalence of MG patients

**Figure 3:**
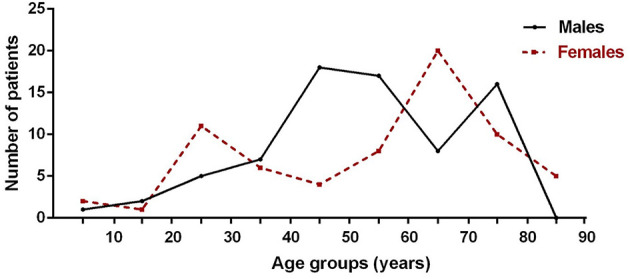
Presence of MG by age of onset and sex (*n* = 118).

In this study, the mean onset age of 118 MG patients was 55.14 ± 17.49 years, and 44.07% were women (Table 1). The incidence was highest in male patients aged 40 to 60 and 70 to 80 and in female patients aged 20 to 30 and 60 to 70 (Figure 3). Myasthenia gravis was more common in the elderly and young women. Of these patients, 33.90% of them (*n* = 40) were diagnosed to be EOMG, 34.75% of them (*n* = 41) were diagnosed to be OMG, and 30.51% of them (*n* = 36) were diagnosed to be TAMG (Table 1). In total, 18.64% of MG patients (*n* = 22) had T2DM.

**Table 1:** Demographic characteristics of MG patients.

**Characteristics**	**Cases (*n* = 118)**
Male: Females	66: 52
Age of onset (years: mean ± sd)	55.14 ± 17.49
**EOMG/LOMG**	
EOMG	40 (33.90%)
LOMG	78 (66.10%)
**OMG/GMG**	
OMG	41 (34.75%)
GMG	71 (60.17%)
Not available	6 (5.08%)
**TAMG/NTAMG**	
TAMG	36 (30.51%)
NTAMG	82 (69.49%)

Demographic characteristics of 118 MG patients.

### 3.2. Risk of MG associated with T2DM

In this study, the objective was to study the effects of T2MD exposure on MG. The risk of MG was higher among T2DM patients than those without T2DM (OR = 1.92, 95% CI: 1.12–3.29, Table 2) in the health examination population. Stratified by sex and age, female and elderly diabetic patients were at greater risk of MG. Compared with women without T2DM, the risk of MG was significantly higher for women with T2DM (OR = 4.25, 95% CI: 1.99–9.05, Table 2). Of all the 22 diabetic MG patients, 17 fell ill with MG several years after the onset of diabetes. The remaining five were diagnosed with diabetes for the first time while hospitalized. In dataset B, diabetic MG patients with T2DM onset time after MG or uncertain onset time of diabetes and their controls were excluded from dataset A. In dataset B, T2DM remained significantly associated with the risk of MG among the female patient group (OR = 2.68, 95% CI: 1.13–6.33, Table 2). Women with diabetes have a 2.68 times higher risk of developing MG than the general population, while the results of other subgroups were not statistically significant.

**Table 2:** Distribution of T2DM and associated risk for MG in datasets A and B.

	**Diagnosed with T2DM**	**Distribution**, ***n*** **(%)**		**Risk for MG, OR (95%CI)**	***P* value**
		**MG patients**	**Matched controls**		
	No	96 (81.36)	526 (89.15)	1 (Reference)	0.018
	Yes	22 (18.64)	64 (10.85)	1.92 (1.12–3.29)	
**Dataset A**	Age < 50 yr				
	No	39 (97.5)	196 (98)	1 (Reference)	0.84
	Yes	1 (2.50)	4 (2.00)	1.25(0.14–11.18)	
	Age ≥ 50 yr				
	No	57 (73.08)	330 (84.62)	1 (Reference)	0.02
	Yes	21 (26.92)	60 (15.38)	1.98 (1.13–3.46)	
	Males				
	No	58 (87.88)	286 (86.67)	1 (Reference)	0.79
	Yes	8 (12.12)	44 (13.33)	0.89 (0.39–2.03)	
	Females				
	No	38 (73.08)	240 (92.31)	1 (Reference)	0.0002
	Yes	14 (26.92)	20 (7.69)	4.25 (1.99–9.05)	
**Dataset B**	Age < 50 yr				
	No	39 (97.5)	196 (98)	1 (Reference)	0.84
	Yes	1 (2.50)	4 (2.00)	1.250 (0.140–11.184)	
	Age ≥ 50 yr				
	No	57 (78.08)	306 (83.84)	1 (Reference)	0.24
	Yes	16 (21.92)	59 (16.16)	1.444 (0.782–2.666)	
	Males				
	No	58 (87.88)	286 (86.67)	1 (Reference)	0.79
	Yes	8 (12.12)	44 (13.33)	0.89 (0.39–2.03)	
	Females				
	No	38 (80.85)	216 (91.91)	1 (Reference)	0.03
	Yes	9 (19.15)	19 (8.09)	2.68 (1.13–6.33)	

Distribution of T2DM and associated risk for MG stratified by sex and age in datasets A and B (The controls were randomly drawn from a population without any AID of the health examination system. Data were analyzed by conditional logistic regression analysis).

Further analysis was made in datasets C and D. Dataset C represented the comparison of MG inpatients and inpatients without any AID. The results of dataset C showed that the risk of MG in female T2DM patients was higher than that of non-diabetic hospitalized patients (OR = 2.07, 95% CI: 1.01–4.25, Table 3). Dataset D represented the comparison of MG inpatients and AID inpatients. The results of dataset D suggested that T2DM patients were at higher risk for MG than those with other AIDs in female patients and the elderly (females: OR = 2.40, 95% CI: 1.19–4.86; elderly: OR = 1.91, 95% CI: 1.09–3.34, Table 3). In a word, diabetic patients had a higher risk of MG, whether compared with ordinary hospitalized patients or patients with other AIDs.

**Table 3:** Distribution of T2DM and associated risk for MG in datasets C and D.

	**Diagnosed with T2DM**	**Distribution**, ***n*** **(%)**		**Risk for MG, OR (95%CI)**	***P* value**
		**MG patients**	**Matched controls**		
**Dataset C**	Age < 50 yr				
	No	39 (97.5)	189 (94.5)	1 (Reference)	0.44
	Yes	1 (2.50)	11 (5.50)	0.44 (0.05–3.51)	
	Age ≥ 50 yr				
	No	57 (73.08)	320 (82.05)	1 (Reference)	0.07
	Yes	21 (26.92)	70 (17.95)	1.66 (0.95–2.89)	
	Males				
	No	58 (87.88)	289 (87.58)	1 (Reference)	0.95
	Yes	8 (12.12)	41 (12.42)	0.97 (0.43–2.19)	
	Females				
	No	38 (73.08)	220 (84.62)	1 (Reference)	0.047
	Yes	14 (26.92)	40 (15.38)	2.07 (1.01–4.25)	
**Dataset D**	Age < 50 yr				
	No	38 (97.44)	171 (91.44)	1 (Reference)	0.23
	Yes	1 (2.56)	16 (8.56)	0.29 (0.04–2.21)	
	Age ≥ 50 yr				
	No	57 (73.08)	324 (83.94)	1 (Reference)	0.02
	Yes	21 (26.92)	62 (16.06)	1.91 (1.09–3.34)	
	Males				
	No	57 (87.69)	277 (86.29)	1 (Reference)	0.76
	Yes	8 (12.31)	44 (13.71)	0.88 (0.40–1.98)	
	Females				
	No	38 (73.08)	218 (86.51)	1 (Reference)	0.02
	Yes	14 (26.92)	34 (13.49)	2.40 (1.19–4.86)	

Distribution of T2DM and associated risk for MG stratified by sex and age in datasets C and D (the controls of dataset C were randomly drawn from all hospitalized patients without any AID. The controls of dataset D were randomly drawn from inpatients diagnosed with AIDs except MG. Data were analyzed by conditional logistic regression analysis).

In all four datasets, only the results of the female group were consistently positive. The greater risk of MG associated with the prevalence of T2DM was more noteworthy in female patients. The female population was stratified by the age of 50. As shown in Table 4, elderly women with diabetes were at a higher risk of MG compared with four different sources of the control group.

**Table 4:** Distribution of T2DM and associated risk for MG in female patients.

**Females Age ≥50 yr**	**Diagnosed with T2DM**	**Distribution**, ***n*** **(%)**		**Risk for MG, OR (95%CI)**	***P* value**
		**MG patients**	**Matched controls**		
Dataset A	No	21 (60)	156 (89.14)	1 (Reference)	0.0001
	Yes	14 (40)	19 (10.86)	4.56 (2.11–9.86)	
Dataset B	No	21 (70)	132 (88)	1 (Reference)	0.02
	Yes	9 (30)	18 (12)	2.87 (1.20–6.8787)	
Dataset C	No	21 (60)	137 (78.29)	1 (Reference)	0.03
	Yes	14 (40)	38 (21.71)	2.25 (1.08–4.70)	
Dataset D	No	21 (60)	141 (82.46)	1 (Reference)	0.005
	Yes	14 (40)	30 (17.54)	2.90 (1.38–6.08)	

Distribution of T2DM and associated risk for MG in female patients aged over 50 years (analyzed by conditional logistic regression analysis).

Furthermore, the characteristics of diabetic and non-diabetic MG patients were observed. The average course of DM in MG patients was 7.64 years by the time of admission. The mean onset age of diabetic MG patients was higher than that of the non-diabetic MG patients (68.23 ± 10.11 vs. 52.15 ± 17.48, *P* < 0.001, Table 5). There was a higher proportion of LOMG clinical type in diabetic MG patients than in non-diabetic MG patients (95.45% vs. 59.38%, *P* < 0.001, Table 5). Of the 17 diabetic MG patients with T2DM onset time before MG, six patients had been tested for antibodies. The AChR antibody was found in 66.67% (*n* = 4) of them, which was not statistically different from that in MG patients without T2DM (87.10%).

**Table 5:** Different characteristics between non-diabetic and diabetic MG patients.

	**MG without T2DM**	**MG with T2DM**	**Available number**	***P***
N	96	22	118	
Gender, females (%)	38 (39.58)	14 (63.64)	118	0.056
Onset age (mean ± sd)	52.15 ± 17.48	68.23 ± 10.11^***^	118	<0.001
LOMG (%)	57 (59.38)	21 (95.45)^***^	118	<0.001
OMG (%)	36 (39.13)	5 (25.00)	112	0.31
TAMG (%)	33 (34.38)	3 (13.64)	118	0.07
Thymoma WHO classification (%)			22	1
A	1 (4.76)	0 (0.00)		
AB	3 (14.29)	0 (0.00)		
B1	5 (22.81)	1 (100.00)		
B2	6 (28.57)	0 (0.00)		
B3	3 (14.29)	0 (0.00)		
B2 + B3	3 (14.29)	0 (0.00)		
Serology (%)			39	0.59
AchR-Pos	27 (87.10)	6 (0.75)		
AchR-Neg	4 (12.90)	2 (0.25)		
Average course of DM	/	7.64 years		
**Laboratory indexes**				
Fasting blood glucose (mean ± sd)	5.21 ± 1.08	7.36 ± 4.21^***^	91	<0.001
Glycated hemoglobin (mean ± sd)	5.70 ± 1.02	6.92 ± 0.99^***^	58	<0.001

Onset age and laboratory indexes were compared by Mann-Whitney U test. Dichotomous variables were compared by the χ^2^ test. Polytomous variables (Thymoma WHO classification) were compared by Fisher exact probability test. *n* = 118; ^***^P < 0.001.

## 4. Discussion

Research has confirmed that a high proportion of abnormalities in glycolipid metabolism has been present in MG patients before treatment with glucocorticoids (16). According to the International Diabetes Federation (IDF) Diabetes Atlas (eighth edition 2017) and the latest Chinese diabetes epidemiological survey, the prevalence of diabetes (20–79 years old) in China was 10.9% (17), which was far lower than that of MG hospitalized patients in this study (24.26%). The probability of MG patients complicated with diabetes was higher than that of the general population. The risk of MG in association with T2DM exposure was explored through an EMR-based, 1:5 matched case–control study. This study illustrated distinct age- and sex-specific associations between T2DM and MG.

In this study, the results of four datasets showed that patients with T2DM had a higher risk of MG than those without T2DM. The sources of the control population of the four datasets were different. Datasets A and B represented the comparison of MG patients and the general population settled in the area. The findings can be extrapolated to the whole population more reliably by selecting controls from the local population without MG. The result of dataset A could only reveal a statistical association between T2DM exposure and MG but not a causal association. For chronic diseases, the time interval between exposure and illness still needs to be considered to determine the cause and effect of this association. In the MG patients of dataset B, the onset of diabetes preceded MG, which is consistent with the sequence of a causal association. The results of dataset B further proved that T2DM might be one of the causative factors of MG. Datasets C and D represented the comparison of MG inpatients and other inpatients, which made better homology and comparability. The results of dataset C demonstrated that MG is associated with diabetes in the female hospitalized population. The results of dataset D further extended the significance of this study. The controls of dataset D were other AID inpatients. The result of dataset D illustrated that MG, but not any other autoimmune disease, was strongly associated with diabetes.

The female population of four different datasets was further stratified by age. The results suggested that T2DM remained significantly associated with the risk of MG in any dataset. Age may be the deciding determinant among female patients as well. As it is well known, the significant asymmetry of AIDs between sexes is thought to be related to sex hormones, sex chromosomes, gut microbiota, and so on (18). There may be differences in etiology according to sex and aging that affect the progression of MG in diabetic patients. Research on related molecular mechanisms is urgently needed.

The mean onset age of diabetic MG patients was significantly higher than that of non-diabetic MG patients, illustrating that the clinical types of diabetic MG patients were mostly LOMG (Table 5). It has been reported that diabetes was more prevalent in LOMG when compared to EOMG (19, 20). There are no differences in different autoantibodies in MG with type 2 diabetes, compared with patients without T2DM. The AChR antibody still takes the overwhelming majority when tested in Chinese patients (21). The lack of positive results may be related to data deficiencies.

A verdict of causation can be safely made only on a sufficient totality of evidence. Several positive criteria support a judgment of causality, including (22, 23) (1) strength; (2) consistency; (3) specificity; (4) temporality; (5) biological gradient; (6) plausibility; (7) coherence; (8) experiment; and (9) analogy. In our study, the risk for MG in T2DM patients was 1.91 to 4.56 times higher than that of groups without T2DM. After identifying the temporal sequence of DM and MG, the results were still positive. The same answer has been reached by different populations with different circumstances. This study should be viewed in the context of its limitations. First, dose–response relationship analyses about the severity of illness and more clinical subgroup analyses, except for age and sex, were not attempted. Second, there may be other unknown confounding factors or mediating variables related to both MG and T2DM, obscuring or exaggerating the relationship between MG and T2DM.

This new finding has led to our further thinking. The concept of diabetic MG may need to be paid more attention to. It may have unique clinical electrophysiology and also clinical and immunological phenotypes distinct from previous subgroup classifications. Previous studies could not explain the relationship between T2DM and the subsequent onset of MG. A protein named advanced glycation end products (AGEs), which are greatly augmented in the early stage of diabetes, might play an important role in this process. As the main ligand of the receptor for AGEs (RAGE) (24), AGEs stimulate inflammatory signal amplification, produce several cytokines and growth factors (25), enhance the proliferation response of CD4(+)CD28(-) T cells (26), and further act in AIDs (27, 28). RAGE mainly acts in pro-inflammatory cellular responses and contributes to the failure of self-tolerance mechanisms (29). MG patients with strong RAGE expression in the thymus tissue had higher levels of AChR-Ab, especially at the disease onset of TAMG (30). In experimental autoimmune MG (EAMG) animal models, increased expression of RAGE and its ligand S100B enhanced T-cell pro-inflammatory responses, affected the Th1/Th2/Th17/Treg cell equilibrium, and upregulated AChR-specific T-cell proliferation (31). S100B-RAGE interaction promotes the production of antibodies against AchRs from the spleen of EAMG (32). The clinical importance of AGEs-RAGE signaling in diabetic MG pathophysiology should be emphasized.

Recently, our group has confirmed that DM aggravated the aberrant humoral immunity in MG patients by promoting differentiation and activation of circulating follicular helper T (Tfh) cells (33). *In vitro*, hyperglycemia increased the expression of inducible costimulatory (ICOS) in Tfh cells *via* the mechanistic target of the rapamycin (mTOR) signaling pathway. This process promoted the differentiation of plasmablasts and IgG secretion. Monitoring humoral immune indicators may be beneficial to patients. The possible immunologic link between MG and T2DM will provide a new strategy for the prevention and treatment of MG.

## 5. Conclusion

Overall, this study has shown a significant association between T2DM and MG, suggesting T2DM may be a risk factor for the onset of MG. Basic experiments to explore relevant molecular mechanisms are needed, but there is no denying that the relationship between T2DM and MG has come to our attention. We hypothesize that a diabetic MG subtype may exist in LOMG patients. Further personalized diagnosis, treatment, and management of diabetic MG patients should be on the agenda.

## Data availability statement

The original contributions presented in the study are included in the article/supplementary material, further inquiries can be directed to the corresponding author.

## Ethics statement

The studies involving human participants were reviewed and approved by Research Ethics Committee of the First Affiliated Hospital of Shandong First Medical University (No: S030). The patients/participants provided their written informed consent to participate in this study.

## Author contributions

R-SD, Y-DL, and FT contributed to the conception and design of this study. X-LL, HL, C-CW, and BY contributed to data acquisition. Y-DL and X-LL organized the database. PZ, C-LY, and TD contributed to the literature search. Y-DL and Y-FL contributed to data analysis and interpretation and verified the underlying data. Y-DL, FT, YL, and R-SD contributed to drafting the manuscript and figures. All authors contributed to the manuscript revision and read and approved the submitted version of the manuscript.
